# The development of a complex intervention in China: the ‘Caring for Couples Coping with Cancer “4Cs” Programme’ to support couples coping with cancer

**DOI:** 10.1186/s12904-015-0062-7

**Published:** 2015-11-23

**Authors:** Qiuping Li, Yinghua Xu, Huiya Zhou, Alice Yuen Loke

**Affiliations:** Wuxi Medical School, Jiangnan University, Wuxi, Jiangsu Province China; Wuxi People’s Hospital, Wuxi, Jiangsu Province China; School of Nursing, The Hong Kong Polytechnic University, Hung Hom, Kowloon, Hong Kong China

**Keywords:** Complex intervention, Medical Research Council (MRC) framework, Spousal caregiver, Couples coping, Cancer

## Abstract

**Background:**

As the primary informal caregiver for cancer patients, spousal caregivers are a population at a high risk of hidden morbidity. The factors impacting couples coping with cancer are complex, and within spousal caregiver-patient dyads the impact is mutual. The aim of this study is to explain the process that led to the development of an acceptable, feasible, and potentially effective ‘Caring for Couples Coping with Cancer “4Cs” Programme’ to support couples coping with cancer as the unit of intervention in China.

**Methods:**

The Medical Research Council’s (MRC) framework for developing and evaluating complex interventions was adopted to develop an intervention for couples coping with cancer. Three steps were taken in the process of developing the 4Cs programme, namely: (1) identifying the evidence base by conducting a series of extensive reviews of the relevant literature and conducting a focus group study; (2) identifying/developing a theory by proposing a preliminary Live with Love Conceptual Framework ‘P-LLCF’ for cancer couple dyads; and (3) modelling the process and outcomes of the 4Cs programme.

**Results:**

The programme that was developed is comprised of six individual sessions to be delivered by the researcher/therapists over 6 weeks. The main contents of the sessions of the 4Cs programme are: primary stressors (section 1); secondary stressors (section 2); dyadic mediator (section 3); dyadic appraisal (section 4); dyadic coping (section 5); and a programme overview (section 6). The emphasis of the programme is on supporting couples coping with cancer. A booklet was developed to provide the participants with easy access to written information about their common concerns in caring for loved ones with cancer.

**Conclusions:**

Guided by the MRC guidelines, a potentially effective, feasible, and acceptable 4Cs programme aimed at supporting couples coping with cancer as a dyad was developed. Future research is needed to pilot and evaluate the feasibility, modelling, and outcomes of this 4Cs programme.

**Electronic supplementary material:**

The online version of this article (doi:10.1186/s12904-015-0062-7) contains supplementary material, which is available to authorized users.

## Background

A complex intervention is an intervention that consists of various components that act independently or inter-dependently [1, 2], and whose function and process are standardised [3]. It is considered beneficial and, indeed, crucial to include both qualitative and quantitative studies in the lengthy process of developing, piloting, evaluating, reporting, and implementing a complex intervention [1].

As a result of early detection and improved treatments for cancer, a large proportion of individuals diagnosed with cancer can expect to live for 5 years after the diagnosis [4]. However the biology of cancer is complex, e.g., survival rates vary across cancer types/staging at diagnosis, and regions according to the level of economic development. For example, a comparative survey on cancer survival rates showed that the 5-year survival rates for breast cancer were 50 % or less in parts of Africa, India, and the Philippines, and over 75 % in Singapore, South Korea, and parts of China. Nevertheless, with the overall trend in cancer survival rates extending beyond 5 years, the burden of care usually falls on family caregivers, particularly on the spouse [5–7]. Given that spousal caregivers are most likely to be the primary caregivers, who are willing to make sacrifices in caring for their partner, they may be especially vulnerable [8, 9]. It has been reported that spousal caregivers experience more fatigue, have less energy, and have more difficulty sleeping than non-spousal caregivers [10]. Spousal caregivers are also said to experience levels of distress as high as or even higher than those felt by the patients themselves [11, 12].

Consequently, the spousal caregivers of cancer patients are at a high risk of developing hidden morbidities according to the World Health Organisation’s definition of the psychological, physical, and social dimensions of health [13]. A review has shown that the spousal caregivers of patients with cancer suffer from a wide spectrum of hidden morbidities, including psychological morbidity (distress, depression, and anxiety), physical morbidity (low levels of physical health, physical functioning, and physical strength), and social morbidity (lower levels of marital satisfaction and social support) [14]. However, spousal caregivers receive little support to perform their vital role of caring for their partner with cancer [15].

The experience of spousal caregivers, however, is complex and relationships are dynamic [16]. The diagnosis and treatment of cancer may pose a strain on the relational dynamics of cancer couples. It can have an impact on the subjective well-being and ability to cope of both the patient and his/her spouse [17]. The findings from a review of how spousal caregiver-cancer patient dyads coped and adjusted revealed that the process of coping with cancer affected both parties, with reciprocal influences and congruence between the spousal caregiver-patient dyads. It also showed that a satisfying pattern of communication between couples was related to lower levels of distress and better marital adjustment [18].

Taking into account the hidden morbidities and relational dynamics of cancer couples, it is concluded that the factors that have an impact on couples coping with cancer are complex and multi-faceted, and that there is a need for a complex intervention to support cancer dyads. To develop a potentially acceptable, feasible, and complex intervention to support cancer couple dyads, guided by the Medical Research Council’s (MRC) framework for developing and evaluating complex interventions [1, 19], the research team conducted a series of separate but interrelated studies beginning in March 2012. This paper presents the development of a complex intervention based on the studies that were undertaken. The aim of this paper is to explain the process that led to the development of a complex intervention, the ‘Caring for Couples Coping with Cancer “4Cs” Programme’ to support couples coping with cancer as the unit of intervention in China, based on the studies that were undertaken.

## Method

The MRC’s framework for developing and evaluating complex interventions was adopted in developing this 4Cs programme [1, 19]. Four phases are indicated in the framework: development, determination of feasibility/piloting, evaluation, and implementation. The development phase involves three steps: identifying the evidence base, identifying/developing a theory, and modelling the process and outcomes. This paper focuses on the first phase in the development of the 4Cs programme. Table 1 outlines the three steps to developing an intervention according to the guidelines of the MRC, and the corresponding elements in developing the 4Cs programme. Ethical approval of the focus group study was obtained from the Human Ethics Committee of the Hong Kong Polytechnic University. Approval for access was obtained from the hospital in Wuxi city in which the study was conducted.

**Table 1:** The three steps to developing a complex intervention according to the MRC and the steps to developing the Caring for Couples Coping with Cancer 4Cs Programme

Steps in the MRC framework for developing a complex intervention	Steps taken to develop the 4Cs Programme
Identifying the evidence base	✓ Conducting a series of extensive reviews of studies related to the spousal caregivers of cancer patients
	✓ Primary research: Conducting a focus group study: the Experiences of Chinese Couples Living with Cancer
Identifying/developing a theory	✓ Proposing a preliminary Live with Love Conceptual Framework (P-LLCF) for cancer couple dyads
Modelling process and outcomes	✓ Developing and presenting the related contents of the 4Cs programme

## Results

This section presents the steps that we took in developing a complex intervention, the 4Cs programme, according to the MRC framework, namely: (1) identifying evidence: evidence was identified from reviews of the literature and a focus group interview study; (2) identifying or developing a theory: a preliminary Live with Love Conceptual Framework (P-LLCF) was proposed; and (3) modelling the process and outcomes of the 4Cs programme. It is worth noting that the extensive literature reviews [14, 18, 20–23], focus group study [24], and a Preliminary Live with Love Conceptual Framework (P-LLCF) for Cancer Couple dyads [23] have been published. Only the main findings of this previous work were re-illustrated in this report for a better understanding of their contribution in the process of developing this 4Cs programme.

### The identified evidence

The first step in developing an intervention in accordance with the MRC framework was to identify the existing evidence through extensive reviews of the literature and by a focus group study.

### Reviews of the literature

To identify evidence of relevance to the subject of spousal caregivers of cancer patients in mainland China, a review of the literature on related studies in China was attempted. Studies focusing on the psychological status, quality of life (QOL), caregiving burden, and social support of family caregivers were identified, but only a few intervention studies focusing specifically on spousal caregivers were found. The conclusion is that, although family caregivers of cancer patients in China have received attention from clinicians and researchers, studies on the subject are still in the stage of infancy [25].

For a better understanding of the phenomenon of spousal caregiving, a series of extensive reviews of the literature related to spousal caregivers of cancer patients was then conducted. These reviews of the literature covered the following aspects: (1) the stress experienced by spouses in caring for cancer patients [21]; (2) the spectrum of hidden morbidities among spousal caregivers of cancer patients [14]; (3) the positive aspects of spousal caregiving for cancer patients [20]; (4) the mutuality of the impact between spousal caregiver-cancer patient dyads [18]; and (5) couple-based interventions for couples coping with cancer [22].

The findings from the previous extensive reviews of the literature [14, 18, 20–23] provided some valuable suggestions on the development of interventions for couples coping with cancer, including: the target population for the interventions should be spousal caregivers and cancer patient dyads; interventions should be provided to the couple as a unit [18]; and there should be a comprehensive dyadic theoretical framework on couples coping with cancer [23] to guide the development of an intervention and outcome measures [22].

These reviews provided a foundation of evidence on the subject of couples coping with cancer, identified gaps in the studies, and offered valuable suggestions on interventions that could be developed focusing on couples coping with cancer. Few couple-based interventions were found that focused specifically on supporting those caring for a spouse with cancer. None of the interventions included in the reviews of the literature evaluated the outcomes of an intervention from the perspective of couples, in terms of their appraisal of the situation, coping strategies, and various health outcome measures, such as QOL and marital satisfaction.

### A primary research study—the experiences of Chinese couples living with cancer

Focus group interviews were conducted among cancer couple dyads [24] to gain a better understanding of Chinese couples coping with cancer, and to explore their experiences, concerns, and needs. The focus group interviews with cancer patients and spousal caregivers led to the identification of four themes and 15 subthemes after a conventional content analysis [24]. The four themes were: communication dynamics, living with changes, negative and positive impacts, and network of support.

Based on the findings, we drew up a preliminary conceptualisation of the couples’ experiences of coping and living with cancer as a whole (Additional file 1: Figure S1). As shown in Additional file 1: Figure S1, the dyadic relationship of cancer couple dyads is conceptualised as an interaction involving communication dynamics, living with changes, and experiencing the negative and positive impacts of coping with cancer. By improving communication and support networks, couples with cancer as dyads will be better able to cope with cancer. The internal interactions of the dyads and their external relationships with peers, relatives, and professional caregivers are represented by a complex pattern of connected themes.

The participants in the study also expressed the need for a couple-based intervention. This study provided insights for healthcare professionals on the daily struggles of couples living with cancer, and on the development of an intervention programme to support these couples. The findings of this primary focus group study not only contributed to the evidence base from the perspective of cancer couples, but also provided us with the information that was needed to choose the constructs to be included in the development of the conceptual framework.

#### The proposed theory

According to the MRC [19], the second step in developing an intervention involves identifying or developing a conceptual framework. It is in this step that a preliminary Live with Love Conceptual Framework (P-LLCF) was proposed [23] for cancer couple dyads. The P-LLCF was developed specifically for cancer couple dyads and had also previously been published [23]. This P-LLCF was developed based on the conceptual frameworks adopted in literature related to the subject of spousal caregiving for patients with cancer, including the Stress and Coping Model (SCM) [26], the Conceptual Framework of the Positive Aspects of Caregiving (CFPAC) [27], the Relationship Intimacy Model (RIM) [28], a Development-Contextual Model of Couples Coping with Chronic Illness (CCCI) [29], and the Cancer Family Caregiving Experience Model (CFCE) [30], and on findings from the focus group study [24].

It was the process of analysing theoretical concepts [31, 32] that guided the development of the P-LLCF. Taking the Stress and Coping Model (SCM) [26] as an example, the SCM provides a conceptual basis for this P-LLCF with regard to the process of coping with stress, and includes the domains of event situation, coping, and outcomes.

The proposed P-LLCF consists of three domains: Event Situation, Dyadic Mediators, and Caregiver-patient Dyads (Additional file 2: Figure S2). As shown in Additional file 2: Figure S2, Event Situation, at the bottom of the conceptual framework, refers to the context and related stressors experienced by cancer couple dyads. The Dyadic Mediators act as ‘leverage’ to balance or off-set the stressors leading to the Dyadic Appraisal, Coping, and Adjustment of the cancer couple dyads at the top of the conceptual framework.

This P-LLCF sheds new light on the study of cancer couple dyads. To our knowledge, ‘the P-LLCF is one of the first conceptual frameworks to specifically focus on a couple’s love in the context of cancer. This P-LLCF has the potential to be useful in developing support programmes and services based on this cancer couple dyads perspective’ [23] (p. E34). The intervention programme guided by this framework can lead to positive outcomes in the caregiving experience of the caregiver-patient dyads, with improvements in dyadic mediators, dyadic coping, dyadic appraisal, and dyadic outcomes throughout the cancer trajectory, helping the couples to ‘Live with Love’. ‘Love in this context is defined as “the active care and concern for the growth to wholeness of the human person”. “Live with Love” was coined with the intention of evoking the deep inner love that couples have for each other’ [23] (p. E33).

#### The developed ‘Caring for Couples Coping with cancer “(4Cs)” Programme’

In the third step of the MRC framework for developing a complex intervention, a ‘Caring for Couples Coping with Cancer “4Cs” Programme’ and education booklet were developed according to the P-LLCF proposed in the second step. A review of the literature on couple-based intervention studies for couples coping with cancer was also conducted to direct the development of the intervention [22].

### Essential components of the 4Cs programme

**Fig. 1:**
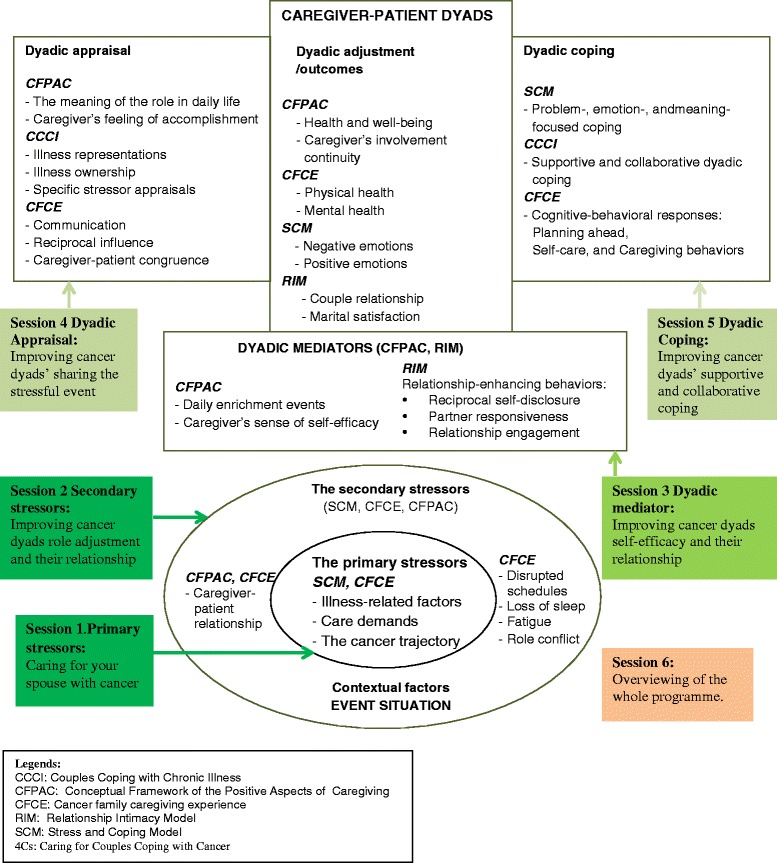
The essential components and focus of the ‘4Cs’ programme developed based on a preliminary Live with Love Conceptual Framework (P-LLCF) for Cancer Couple Dyads

The essential components and focus of the 4Cs intervention programme were developed based mainly on the P-LLCF for Cancer Couple Dyads (Fig. 1). The 4Cs programme takes into account the three domains of the P-LLCF: Event Situation, Dyadic Mediators, and Caregiver-patient Dyads. This programme consists of six weekly sessions, with each session lasting for 90 min. The main contents of the sessions of the 4Cs programme are: primary stressors (section 1); secondary stressors (section 2); dyadic mediator (section 3); dyadic appraisal (section 4); dyadic coping (section 5); and a programme overview (section 6). The session titles, aims/contents, and approaches adopted are listed in Table 2.

**Table 2:** Title, aims/contents, and approaches of the programme sessions

Session number, main focus, and title	Aims/contents	Approaches		
		PE	ST	CBT
1. Primary stressors: Caring for your spouse with cancer	- To present key strategies relating to *illness-related factors* and *care demand*;	√	√	
	- To help cancer dyads to gain more confidence in responding to the physical and psychosocial issues of both patients’ and caregivers;	√	√	√
	- To outline the services available from the cancer caring team and other support services.	√		
2. Secondary stressors: Improving the role adjustment of cancer dyads and their relationship	- To facilitate the role conflict and adjustment of cancer dyads by providing them with verbal and written information about typical aspects and common issues associated with their *roles* as an individual cancer patient and/or a spousal caregiver, and as a dyad within the relationship;	√		
	- To support cancer dyads by focusing specific attention on their needs, including having enjoyable experiences, getting enough sleep, following a healthy diet, getting enough exercise, and having a good relationship;	√	√	
	- To reinforce the role of the cancer care service.	√		
3. Dyadic mediator: Improving the self-efficacy of cancer dyads and their relationship	- To help cancer dyads to appreciate the daily enrichment events;	√	√	√
	- To elevate the dyads’ sense of self-efficacy;	√	√	√
	- To encourage the cancer dyads to practise relationship-enhancing behaviours, including self-disclosure and being responsive to one’s partner;	√	√	√
	- To improve cancer-related communication between couples by educating them to take the view of ‘hoping for the best, preparing for the worst’.	√	√	
4. Dyadic appraisal: Improving the sharing by cancer dyads of stressful events	- To help cancer dyads to acknowledge the meaning of their role in daily life and to give caregivers a feeling of accomplishment;	√		√
	- To facilitate the sharing by dyads of stressful events by helping them to appraise presentations of the illness, the ownership of the illness, and specific stressors;	√	√	√
	- To help the dyads to understand strategies for maintaining a good relationship: ‘communication’, ‘reciprocal influence’, ‘caregiver-patient congruence’.	√	√	
5. Dyadic coping: Improving supportive and collaborative coping by cancer dyads	- To improve supportive and collaborative coping by cancer dyads by facilitating meaning-focused coping strategies for benefit finding, benefit reminding, adaptive goal processes, reordering priorities, and infusing ordinary events with positive meaning;	√	√	√
	- To promote self-care on the part of spousal caregivers by encouraging them to plan ahead and enhance their physical and mental health by promoting regular ‘time outs’, enjoyable experiences, enough sleep, a healthy diet, exercise, and advice on relaxation strategies.	√	√	√
6. Overview of the whole programme	- To help cancer dyads to review the main contents of the programme and to address any problems that they might have.	√	√	√

*CBT* cognitive-behavioural therapy, *PE* psycho-educational, *SK* skills training

### Intervention approaches

The approaches that will be adopted in this couple-based intervention programme are Psycho-education (PE), Skills Training (ST), and Cognitive Behavioural Therapy (CBT). Psycho-education (PE) is a professionally delivered treatment modality that integrates and synergises psychotherapeutic and educational interventions [33]. The care recipients, including both the patient and spousal caregiver, are considered partners with the healthcare provider in the intervention relationship. The psycho-education is based on the premise that the more knowledgeable the care recipients are about the related event, the more positive the health-related outcomes will be for care recipients [33]. In this programme, psycho-education is defined as protocols whose primary focus is to provide information on primary and secondary stressors, including illness-related factors (such as symptom management); care demands (such as physical aspects of patient care); the role conflicts of patients or caregivers; as well as the caregiver-patient relationship.

Skills training (ST) is defined as ‘the teaching of specific verbal and nonverbal behaviours and the practising of these behaviours by the patient’ [34]. In this programme, skill training is defined as protocols that focus primarily on the development of problem-, emotion- and meaning-focused coping skills, the self-care behaviours of the caregivers, and the relationship-enhancing strategies of the cancer dyads.

Cognitive Behavioural Therapy (CBT) is a psychotherapeutic approach that helps patients (cancer dyads) understand the thoughts and feelings that influence behaviours. CBT is commonly used to treat a wide range of disorders, including depression and anxiety [35]. CBT is based on the idea that a person’s thoughts and feelings play a fundamental role in his/her behaviour. The goal of CBT is to teach patients (cancer dyads) that while they cannot control every aspect of the world around them, they can take control of how they interpret and deal with things in their environment [35].

### Education booklet

A guidebook for the spousal caregivers titled ‘Live with Love: Hope for the best, prepare for the worst’ was developed based on reviews of the literature, and the preliminary conceptual framework will be used to complement the group intervention programme. The guidebook will provide spousal caregivers with easy access to written information on common concerns about caring for a partner with cancer. The main contents of the booklet are shown in Table 3.

**Table 3:** Contents of the booklet: Live with Love - Hope for the best, prepare for the worst

Title	Contents
Primary stressors^a^	✓ Helping with medications
❖ Taking care of your spouse with cancer	✓ Providing hygiene car
	✓ Assisting with eating and drinking
	✓ Help with other ‘technical’ care
	✓ Use of special equipment
	✓ Dealing with common symptoms
	- Pain (including concern about opioids and hastening death); Nausea; Constipation; Breathlessness; Fatigue; Delirium
	✓ Emotional care
	✓ Spiritual care at the end of life
	✓ How much should patients be told about their illness?
	✓ Available cancer care resources
Secondary stressors^b^	✓ The Role of the Caregiver
❖ Being a caregiver—what is it about?	✓ Helping to Manage Your Loved One’s Treatment
	✓ Helping Your Loved One With Practical Matters
	✓ Providing Emotional Support
	✓ Caregiving Under Difficult Circumstances
	✓ Taking Care of Yourself
	- Staying Healthy
	- Getting Emotional Support
	- Getting Help With Caregiving Responsibilities
	- Maintaining hope when the situation seems hopeless
	- Feeling overwhelmed? It’s time to relax!
	- Taking a break
Dyadic mediator^c^	✓ Sense of self-efficacy
❖ Caring for your relationships	✓ Reciprocal self-disclosure
	✓ Partner responsiveness
	✓ Relationship engagement
	✓ Family meetings
	✓ Your relationship with the person you are caring for
	✓ Involving children
	✓ Your relationship with family and friends
Dyadic appraisal^d^	✓ The meaning of their role in daily life
❖ Sharing the stressful event	✓ Caregivers’ feeling of accomplishment;
	✓ The illness representations
	✓ Illness ownership
	✓ Specific stressors
	✓ Communication
	✓ Reciprocal influence
	✓ Caregiver-patient congruence
Dyadic coping^e^	✓ Problem-, emotion and meaning-focused coping
❖ Improving supportive and collaborative coping	- Benefit finding
	- Benefit reminding
	- Adaptive goal processes,
	- Reordering priorities
	- Infusing ordinary events with positive meaning
	✓ Cognitive-behavioural responses
	- Planning ahead
	- Self-care
	- Caregiving behaviours

^a^Primary stressors: refer to factors related to the patient’s illness, such as the stage of the cancer, the patient’s physical health, care demands (dependency), and the cancer trajectory

^b^Secondary stressors: consist of role conflict, the caregiver-patient relationship, schedule disruptions, loss of sleep, fatigue, and contextual factors

^c^Dyadic mediator: act as “leverage” to balance or off-set the stressors leading to the dyadic appraisal, coping, and adjustment of the cancer couple dyads. It includes the following components: “daily enrichment events”, “caregiver’s sense of self-efficacy”, relationship-enhancing strategies, e.g. reciprocal self-disclosure, partner responsiveness, and relationship engagement

^d^Dyadic appraisal: refers to the components and representation of the illness, illness ownership, and whether the couple shared the stressors

^e^Dyadic coping: is conceptualized as a continuum of couple involvement ranging from the non-involvement of the spouse, that the patient perceives that he or she is alone in coping with the stressful event, to the over-involvement of the spouse, that the patient perceives the spouse as controlling, in that the spouse dominates the actions of the ill partner by taking charge and telling the partner what to do

### Outcome measures

All of the outcome measures that were included are established instruments with good reliability and validity. The outcome measures were selected based on the constructs of the ‘caregiver-patient dyads’ of the P-LLCF, and are intended to be measured at baseline (T0), after the completion of the 4Cs programme (T1), and three months (T2) after the completion of the programme. These outcome measures include the following items: couples’ self-efficacy, dyadic coping strategies, communication, physical and mental health, depression, benefit finding, and marital satisfaction. The correlation of the outcome variables with components included in the P-LLCF is summarised in Table 4.

**Table 4:** Correlating the outcome measures of the 4Cs programme with components in the P-LLCF^a^

Outcome variables	Instruments & source	Correlation with components in the P-LLCF
Self-Efficacy	The 12-item Cancer Behaviour Inventory (CBI-B) [36]	- Dyadic mediators: caregivers sense of self-efficacy (CFPAC)^a^ [27]
		- Dyadic outcomes: caregivers’ involvement continuity (CFPAC)^a^ [27]
Communication	The 15-item Cancer-Related Communication Problems within Couples Scale (CRCP) [39]	- Dyadic appraisal: communication, reciprocal influence, and caregiver-patient congruence (CFCE)^a^
		- Dyadic outcomes: couple relationship (RIM)^a^ [28]
Dyadic coping strategies	The 37-item Dyadic Coping Inventory (DCI) [37, 38]	Dyadic coping
		- Problem-, emotion-, and meaning-focused coping (SCM)^a^ [26]
		- Supportive and collaborative dyadic coping (CCCI)^a^ [29].
Physical and mental health	The Medical Outcomes Study 12-item short form (MOS SF-12) (version 2) [40]	Dyadic outcome: physical and mental health (CFCE)^a^ [30]
Depression	The 14-item Hospital Anxiety and Depression Scale (HADS) [41]	Dyadic outcome: negative outcomes (SCM)^a^ [26]
Benefit-Finding	The revised 17-item Benefit-Finding Scale (BFS) [42]	Dyadic outcome: positive outcomes (SCM)^a^ [26]
Marital Satisfaction	The 14-item Revised Dyadic Adjustment Scale (RDAS) [43, 44]	Dyadic outcome: marital satisfaction (RIM)^a^ [28]

^a^ *P-LLCF* preliminary-live with love conceptual framework, *CFPAC* conceptual framework of the positive aspects of caregiving, *CFCE* cancer family caregiving experience, *RIM* relationship intimacy model, *SCM* stress and coping model, *CCCI* couples coping with chronic illness

The self-efficacy and involvement continuity (physically or psychologically prepared to take care of the patient) of the caregivers will be evaluated using the 12-item Cancer Behaviour Inventory (CBI-B) [36]. The 37-item Dyadic Coping Inventory (DCI) will be used to assess dyadic coping as perceived by (1) each partner about their own coping, (2) each partner’s perception of the other’s coping, and (3) each partner’s view of how they cope as a couple [37, 38]. The 15-item Cancer-Related Communication Problems within Couples Scale (CRCP) will be used to measure communication between the couples, reflecting the dyadic communication and the couples’ relationship. It has been stated that ‘Open communication is the premise and healthiest form of cancer-related communication, and serves as an indicator of better psychological state and marital relationship’ [39] (p. 783).

The couples’ QOL in terms of physical and mental health, depression, benefit finding, and marital satisfaction will be assessed using the Medical Outcomes Study 12-item short form (MOS SF-12) (version 2) [40], the 14-item Hospital Anxiety and Depression Scale (HADS) [41], the 17-item revised Benefit-Finding Scale (BFS) [42], and the 14-item Revised Dyadic Adjustment Scale (RDAS) [43, 44]. The seven instruments and the source of the instruments are summarised in Table 4.

Information on the demographics and characteristics of both the patients and their spousal caregivers will be collected at baseline. The outcome measures will be completed separately by the spousal caregivers and the cancer patients at baseline, immediately after the intervention, and three months after the intervention. However, health professionals should be cautioned to be selective and consider the potential burden for participants when completing an extensive instrument. Nurses in the oncology unit will assist those who require help completing the questionnaire.

## Discussion

Guided by the MRC framework for developing complex interventions [1, 19], this is a report on the development of the 4Cs intervention programme, which adopts the three steps of identifying the evidence base, identifying/developing a theory, and modelling the processes and outcomes.

According to the guidance provided by the MRC [1, 19], ‘Best practice is to develop interventions systematically, using the best available evidence and appropriate theory, then to test them using a carefully phased approach...’ [1] (p. 980). This is the process that was adopted in developing the 4Cs programme. Given that this 4Cs programme was developed based on the preliminary Live with Love Conceptual Framework (P-LLCF), and that the P-LLCF was developed according to the extensive existing evidence, including findings from reviews of the literature and interviews with cancer couples, this 4Cs programme should be an acceptable, feasible, and effective programme.

This programme was designed to consist of six sessions, each with a different focus. According to the P-LLCF [23], there are direct and indirect interrelationships among the three domains of Event Situation, Dyadic Mediators, and Caregiver-patient Dyads. The same relationships may exist among the three constructs of dyadic appraisal, dyadic coping, and dyadic adjustment in the domain of caregiver-patient dyads. Thus, it can be inferred that these components of the 4Cs programme, which were developed based on the P-LLCF, act both independently and inter-dependently. For instance, the section that focuses on primary stressors can act independently as a simple intervention to benefit couples coping with cancer, while also acting inter-dependently with other sections to support outcomes for couples. This 4Cs programme is considered a complex intervention, since it contains various components [2] and these components act both independently and inter-dependently [45].

While conducting a qualitative study alongside a quantitative study in a randomised controlled trial remains uncommon [45], it is recognised that a mixture of methods incorporating both qualitative and quantitative approaches during the process of developing, evaluating, and implementing a complex intervention is needed. It is highly recommended that in an RCT study of the 4Cs programme, a qualitative approach such as a focus group study should be undertaken as part of the evaluation. This qualitative study should include both couples who adhere to the intervention programme and those who drop out or do not participate in the programme, to gain a better understanding of the degree to which the programme is accepted, the reasons for this, and the barriers to participating in the programme [1].

The 4Cs couple-based intervention programme was developed in accordance with the P-LLCF [23], incorporating the various domains and constructs that were depicted. The intervention programme is intended to facilitate positive dyadic adjustment/outcomes among cancer couples in their journey of coping. The outcome measures include the couples’ self-efficacy, dyadic coping strategies, communication, physical and mental health, depression, benefit finding, and marital satisfaction (Table 4). It is crucial that the intervention programme be piloted and evaluated, and the outcomes tested, before a randomised control trial of the intervention programme is implemented in clinical settings as directed by the MRC framework [19]. It is worth noting that the phases and steps in the updated MRC framework [19] are no longer linear, which gives better opportunities to redevelop the intervention if needed after the pilot study.

## Limitations

Given that this is the first development of a complex intervention for cancer couple dyads in China, the acceptability of the procedures of the programme, and the recruitment and retention of participants to achieve the proposed number of participating dyads, remain uncertain.

## Recommendations for future research

Following the phase of developing a complex intervention, there remains the process of determining its feasibility/piloting, and evaluating and implementing the intervention as prescribed by the guidance given by the MRC [1]. Before implementing a complex intervention, the intervention needs to be tested for feasibility/piloting, as well as to be evaluated. It is recommended that a pilot study be conducted in the next phase to evaluate the feasibility of the 4Cs programme according to the MRC guidance. The main contents of the prospective pilot study are described below. They include the prospective trial design, prospective participants, prospective study settings, delivery of the intervention, and quality assurance.

### Prospective trial design

A mixed-methods study that includes qualitative and quantitative approaches is planned. Before undertaking a full-scale randomised controlled trial (RCT) intervention study to deliver and evaluate the 4Cs programme, a pre-post pilot trial will be conducted in the second phase of the MRC framework to test the feasibility of the 4Cs programme, including the acceptability of the procedures, the validity, reliability, and feasibility of the instruments, the recruitment and retention of participants, and the identification of the appropriate sample size.

### Prospective participants

The target population of the complex intervention will be married couples in which one of the partners has been diagnosed with cancer and the spouse is the primary caregiver. The relationship of the couples will be stratified according to the quality of their relationship prior to the cancer experience, i.e., infatuated love, (un)consummated love, companionate love, and romantic love. To achieve this, a brief interview with couples on the quality of their relationship prior to the cancer experience will be conducted simultaneously during the period of collecting the baseline data. Stratifying the sample according to different cancer types and/or cancer stages also deserves further consideration when designing the study. The criteria for inclusion in the study are: (1) Chinese married couples (aged 18 years and older); (2) who live in Wuxi city; (3) one of the pair of whom has been diagnosed with cancer and who has a life expectancy of at least six months (4) where the spouse is the primary caregiver for the partner with cancer; (5) and both of whom have agreed to take part in the study. The spousal caregiver is defined and identified by the cancer patient as his or her married partner and primary source of physical and emotional support since the diagnosis of cancer. Cancer couples will be excluded if the spousal caregiver is unable to care for himself/herself due to chronic illness, or suffers from a serious physical or mental illness, including cancer.

### Prospective study settings

This study will be conducted in an oncology hospital in Wuxi city, Jiangsu, China. The oncologists in the hospital will be responsible for screening couples in accordance with the inclusion criteria. Couples who meet the eligibility criteria will be approached in oncology wards, and will be given an explanation of the intervention programme and the purpose of the research. Only those couples who sign a consent form indicating their willingness to participate in the study will be included.

### Delivery of intervention

The face-to-face group intervention will be delivered by the researcher/therapist and by nurses who have been provided with extensive training on the intervention programme. The education sessions will be semi-structured, with a mix of didactic instruction (used sparingly) and group sharing and interactions. Sufficient time for questions, comments, clarifications, and dialogue will be an essential feature of each session. It is anticipated that four to six cancer dyads will be included in each programme.

In this intervention programme, the therapist will actively stimulate perspective taking, cognitive restructuring, and behavioural exercises. The therapist and one of the researchers of this study is a medical doctor who treats cancer patients and is also qualified as a psycho-counsellor in mainland China.

### Quality assurance

Strategies will be implemented to ensure that the protocols of the intervention are adhered to, and the intervention will be provided in a uniform manner to ensure treatment fidelity. These strategies will include training nurses in the intervention and protocol; writing a detailed outline of the intervention; audio-taping randomly selected sessions for quality checks; and holding a monthly discussion meeting among the members of the research group.

## Conclusion

A potentially acceptable, feasible, and effective Caring for Couples Coping with Cancer “4Cs” Programme was developed adopting the guidelines of the MRC framework for developing and evaluating complex interventions. This was done with supporting evidence from numerous reviews of the relevant literature, the findings of a focus group study on cancer couple dyads, and a proposed preliminary Live with Love Conceptual Framework (P-LLCF). Future research is needed to pilot and evaluate the feasibility, modelling, and outcomes of this 4Cs programme.

## Additional files

Additional file 1: Figure S1. A preliminary conceptualization of the overall experiences of couples living and coping with cancer. (DOC 225 kb) Additional file 2: Figure S2. A preliminary Live with Love Conceptual Framework (P-LLCF) for Cancer Couple Dyads. (DOC 98 kb)

## Abbreviations

“4Cs”: Caring for Couples Coping with Cancer
CBT: Cognitive Behavioural Therapy
MRC: The Medical Research Council
PE: psycho-education
P-LLCF: Preliminary Live with Love Conceptual Framework
ST: skills training
