# Supplementary material for: The development of a complex intervention in China: the ‘Caring for Couples Coping with Cancer “4Cs” Programme’ to support couples coping with cancer
Source: BMC Palliat Care. 2015 Nov 23;14:64. doi: 10.1186/s12904-015-0062-7 (PMC4657345; doi:10.1186/s12904-015-0062-7)
Supplement: Additional file 1: Figure S1. — A preliminary conceptualization of the overall experiences of couples living and coping with cancer. (DOC 225 kb) [file 12904_2015_62_MOESM1_ESM.doc]

**Dyadic relationship**

**of the couple**

**Communication dynamics**

Expression of appreciation

Compelling need for information

Information censorship and the two-sided face

**Living with changes**

Change in roles

Change in marital relationship

Change in life plan

Change in social activities

**Negative &**

**positive impacts**

Side-effects of chemotherapy

Caregiver burden

Reciprocal caring / support

Positive perspective and hope

**Network …**

**… of …**

**… Support**

Family Support

Comrades in arms against cancer

Support from

healthcare professionals

Governmental support

**Figure S1. A preliminary conceptualization of the overall experiences of couples living and coping with cancer**
