# Supplementary material for: The development of a complex intervention in China: the ‘Caring for Couples Coping with Cancer “4Cs” Programme’ to support couples coping with cancer
Source: BMC Palliat Care. 2015 Nov 23;14:64. doi: 10.1186/s12904-015-0062-7 (PMC4657345; doi:10.1186/s12904-015-0062-7)
Supplement: Additional file 2: Figure S2. — A preliminary Live with Love Conceptual Framework (P-LLCF) for Cancer Couple Dyads. (DOC 98 kb) [file 12904_2015_62_MOESM2_ESM.doc]

**Dyadic appraisal**

***CFPAC***

- The meaning of the role in daily life

**- Caregiver’s feeling of accomplishment**

***CCCI***

**- Illness representations**

**- Illness ownership**

**- Specific stressor appraisals**

***CFCE***

**- Communication**

**- Reciprocal influence**

**- Caregiver-patient congruence**

**CAREGIVER-PATIENT DYADS**

**Dyadic adjustment**

**/outcomes**

***CFPAC***

**- Health and well-being**

**- Caregiver’s involvement continuity**

***CFCE***

**- Physical health**

**- Mental health**

***SCM***

**- Negative emotions**

**- Positive emotions**

***RIM***

**- Couple relationship**

**- Marital satisfaction**

**Dyadic coping**

***SCM***

**- Problem-, emotion-, and meaning-focused coping**

***CCCI***

**- Supportive and collaborative dyadic coping**

***CFCE***

**- Cognitive-behavioral responses: Planning ahead,**

**Self-care, and Caregiving behaviors**

***RIM***

Relationship-enhancing behaviors:

- Reciprocal self-disclosure
- Partner responsiveness
- Relationship engagement

**DYADIC MEDIATORS (CFPAC, RIM)**

***CFPAC***

- Daily enrichment events

- Caregiver’s sense of self-eﬃcacy

**The secondary stressors**

(SCM, CFCE, CFPAC)

**The primary stressors**

***SCM, CFCE***

- Illness-related factors

- Care demands

- The cancer trajectory

***CFPAC, CFCE***

- Caregiver-patient relationship

***CFCE***

- Disrupted schedules

- Loss of sleep

- Fatigue

- Role conflict

**Contextual factors**

**EVENT SITUATION**

**Legends:**

**CCCI:** Couples Coping with Chronic Illness

CFPAC: Conceptual Framework of the Positive Aspects of Caregiving

**CFCE:** Cancer family caregiving experience

**RIM:** Relationship Intimacy Model

**SCM:** Stress and Coping Model

**Figure S2.**

A preliminary Live with Love Conceptual Framework (P-LLCF) for Cancer Couple Dyads
